# CoMeta: Classification of Metagenomes Using *k*-mers

**DOI:** 10.1371/journal.pone.0121453

**Published:** 2015-04-17

**Authors:** Jolanta Kawulok, Sebastian Deorowicz

**Affiliations:** Institute of Informatics, Silesian University of Technology, Gliwice, Poland; Albert Einstein College of Medicine, UNITED STATES

## Abstract

Nowadays, the study of environmental samples has been developing rapidly. Characterization of the environment composition broadens the knowledge about the relationship between species composition and environmental conditions. An important element of extracting the knowledge of the sample composition is to compare the extracted fragments of DNA with sequences derived from known organisms. In the presented paper, we introduce an algorithm called CoMeta (Classification of metagenomes), which assigns a query read (a DNA fragment) into one of the groups previously prepared by the user. Typically, this is one of the taxonomic rank (e.g., phylum, genus), however prepared groups may contain sequences having various functions. In CoMeta, we used the exact method for read classification using short subsequences (*k*-mers) and fast program for indexing large set of *k*-mers. In contrast to the most popular methods based on BLAST, where the query is compared with each reference sequence, we begin the classification from the top of the taxonomy tree to reduce the number of comparisons. The presented experimental study confirms that CoMeta outperforms other programs used in this context. CoMeta is available at https://github.com/jkawulok/cometa under a free GNU GPL 2 license.

## Introduction

Comprehensive and complete analysis of the microbes’ genomes, performed in their original environment, usually called metagenomics [1] or environmental and community genomics, became a popular field of research in recent years. Its origins can be found in the work of Pace *et al.*[2], in which the first proposal for cloning the environmental DNA by Polymerase Chain Reaction (PCR) to explore the diversity of ribosomal RNA sequences was formulated. In metagenomics, the isolation and culture of organisms is unnecessary. Therefore, it is possible to investigate the species that previously have been usually neglected due to the lack of laboratory-grown cultures. Moreover, a large number of unknown enzymes and metabolic capabilities are encoded in the genomes of uncultured species. Ultimately, metagenomics allows for discovering thousands of new microorganisms and their potentially useful functions [3, 4].

Metagenomic analyzes can help in solving numerous practical challenges in medicine, engineering, agriculture, and ecology [5]. Currently, many projects are carried out which are aimed at understanding biocenosis coming from various environments, such as soil [6, 7], water (i.e., groundwater [8], seawater [9, 10], rivers [11]), or places with extreme conditions, like hot springs and mud holes in solfataric fields [12], glacier ice [13], or Antarctic desert soil [14]. The probes are also collected from other organisms, for example from rumens of buffalo [15] or cow [16].

The fact that human organism carries a hundred times more bacterial genes than our inherited human genome was the main reason for growing interests in the microorganisms living in the human body [17–19]. The main aim of the Human Microbiome Project [20], started in 2009, lies in characterizing the human microbiome communities found at several different sites in the human body, including nasal passages, oral cavities, skin, gastrointestinal, and urogenital tracts. Furthermore, the project is aimed at analyzing the role of these microbes in human health and disease.

### Metagenomic processing

The metagenomic analysis is a multi-stage process [4, 21, 22]. First, the genetic material is isolated from the environmental sample containing a mixture of various types of microorganisms. Subsequently, the DNA material is extracted and sequenced. Finally, the reads (short fragments of genomes obtained in sequencing) are binned and annotated.

In the recent decade, the DNA sequencing methods were becoming cheaper and faster. The first method for sequencing was invented by Sanger [23] in 1977, and it dominated for almost two subsequent decades. In spite of many improvements proposed to this technique, it is inferior to the recent methods, referred to as Next Generation Sequencing (NGS) [24]. The most popular among them are the 454/Roche and Illumina/Solexa systems, and nowadays they are extensively applied to the analysis of metagenomic samples [21]. For example, the 454 sequencing has been used to study the metagenomes contained in kefir grains [25], waste water [26], whereas the sequences of infant gut [27] or Cystic Fibrosis Lungs [28] metagenomes have been sequenced with Illumina. In a single experiment, the 454/Roche sequencers produce millions of long reads (600–900 bp), while the Illumina/Solexa sequencers deliver hundreds of millions of shorter reads (36–200 bp).

### Classification of metagenomic data

The sequencing results in obtaining a huge set of reads coming from the genomes of organisms living in the investigated environment. As it was mentioned earlier, an important aim of the metagenomic study is to determine qualitative and quantitative composition of the environmental sample, which is achieved by solving two important tasks, namely binning and annotation. The latter requires classification of the reads to a set of known sequences. The reads may be compared with annotated sequences stored in a number of databases (e.g., GenBank [29]), and associated with a species or a gene function. In general, the questions raised are: “who is there?”, “how much of each?”, and “what are they doing?”. The answers to the first two questions may be obtained relying on taxonomic classification, while the third one can be answered using functional classification.

During the study of the environmental community, the obtained reads derived from a set of various organisms are assigned to taxa. The assignment may be either independent or dependent on the taxonomy. In the latter case, the reads are directly assigned to taxa on the basis of the reference sequences, where the taxon can range from the superkingdom to the species rank. During the taxonomy independent analysis, the reads are grouped into operational taxonomic units (OTUs) based on their similarity to each other in the sample. OTU is usually delineated with a 3% sequence dissimilarity, which corresponds to the taxonomical rank of species [30, 31]. Obviously, the acceptance threshold may be set to a different value [32]. Using the taxonomy dependent analysis, OTUs can be assigned to taxonomic names. In a single habitat, the organisms belonging to various groups appear together. Even though a microbial probe contains microbial eukaryotes, bacteria, archaea, and also viruses, the metagenomic study is primarily focused on the prokaryotic species. Moreover, sequencing of eukaryotic DNA is unprofitable due to the large genome size and low gene coding densities. Therefore, in some studies, the eukaryotic cells are eliminated by filtering the samples [10].

There are several computer programs for read-to-taxa classification. They can be separated into two main groups, namely composition-based and similarity search methods. Using the former, reference sequence features are first extracted and subsequently compared, whilst using the latter, the reads are compared to some reference sequences. The hybrids of these two approaches may also include elements of phylogenetic analysis.

The composition-based methods follow the three-stage strategy [33–38]: 1) machine learning-based modeling of features extracted from reference sequences (e.g., distribution of short nucleotide subsequences, *k*-mers); 2) modeling of the unknown set of reads (performed in the same way as for the set of reference sequences); 3) comparison of the reads and reference sequences models to assign taxonomic ranks for each read. Among the machine learning methods, it is worth to mention the interpolated Markov models [34], support vector machines (SVMs) [37, 38], *k*-nearest neighbors [35] or naive Bayesian classifier [36]. For SVMs, training from large datasets may be problematic, however the training set can be effectively selected using various techniques [39–41].

The similarity search methods rely on the sequence homology. They use a database, containing nucleotide or protein reference sequences. For detecting remote homologies, it is better to use the protein sequences, as they are more well-conserved across greater evolutionary distances. However, in order to use the protein database, the reads have to be translated into amino acid sequences. Taking into account all three possible start sites of encoding amino acids on the both strands (the main sequence and its reverse-complement counterpart), each read has to be translated in all six reading frames, which negatively influences the computation time. In addition, the reads with non-coding DNA cannot be processed by such translation-to-protein method.

In most cases, the similarity search methods employ BLAST to obtain alignments of reads to a reference sequences set. Subsequently, these alignments are used for taxonomic classification. Some programs, like MEGAN [42], MTR [43], SOrt-ITEMS [44], CARMA3 [45], use the lowest common ancestor (LCA) algorithm for assigning the taxonomic labels. After performing the BLAST search for each read, the BLAST hits, whose bit scores are above the threshold, are selected for further analysis. LCA is computed for all species that were reported by best BLAST hits for a read. If BLAST hits are ambiguous (the hits are similar for reference sequences derived from different species), then the read is assigned to a higher taxonomic level.

Furthermore, the marker genes can also be used to facilitate reads classification. These genes help to identify a particular species, e.g., 16S rRNA occurs in the prokaryote genomes. MG-RAST [46] relies on the chloroplast, mitochondrial, and ACLAME (including mobile genetic elements) databases. MetaPhyler [47] uses 31 phylogenetic marker genes as the taxonomic references. One of CARMA3 variants [45] and Treephyler [48] use hidden Markov models (instead of BLAST) to search for the homologies against the Pfam database—protein domains contained in the Pfam are here used as the markers.

As discussed earlier, the composition-based classification methods compare the *k*-mer distribution of a read with those which come from different taxa. In the FACS [49] program, instead of determining the full distribution of *k*-mers, their appearance in a reference sequence is taken into account (1 if a *k*-mer from a read appears in a reference sequence, 0 otherwise). FACS can be regarded as a similar search method, because it aligns the reads to the reference sequences, represented by *k*-mers indexed using the Bloom filters. The original FACS algorithm was implemented in the Perl language, but the latest version has been reimplemented in C (available at https://github.com/SciLifeLab/facs). Actually, the new version is not intended for metagenomic data classification, but it checks how many reads might be contaminated in a particular sample.

The Livermore Metagenomics Analysis Toolkit (LMAT) also maps *k*-mers without using information about their positions and quantity [50]. When constructing a *k*-mer database, each canonical *k*-mer (i.e., the *k*-mer or its reverse complement, if the latter is lexicographically smaller), derived from the reference sequence, is assigned to a group of reference sequences which contain that *k*-mer. Hence, the *k*-mers are grouped together in such a way that each group contains those *k*-mers which occur in every reference sequence in the group and does not occur in any sequence outside the group. LMAT, like the programs discussed earlier, also computes the LCA—the created groups are linked together in a taxonomic tree. During classification, the canonical *k*-mers of each read are compared to the *k*-mers located in every group. The similarity score is increased for each matching *k*-mer, and cumulated for the whole taxon. Similarly to other LCA-employing methods, in case of conflicts (i.e., situations, in which the scores for several taxa are high and identical) the read is classified to the level above. This helps in selecting the most specific taxonomic label, whose lineage has no conflicts with another taxonomic label.

Very recently, the Kraken algorithm [51] using the *k*-mer indexing scheme similar to LMAT, has been proposed. These methods differ, however, in classification and database construction strategy. In the algorithm used in Kraken, each *k*-mer from a reference sequence stores the taxonomic ID number of the *k*-mers’ LCA values. Like in LMAT, the Kraken database contains the *k*-mers in the canonical representation. However, these *k*-mers are first sorted according to the minimizer, a very popular idea in recent years in bioinformatics [52–54], (i.e., the lexicographically smallest *M*-mer in each *k*-mer), and the *k*-mers containing the same minimizer are sorted in the lexicographical order in the database. This strategy substantially accelerates the queries. A taxonomic node cumulates points for every match of a *k*-mer extracted from the given read. The read is classified to the node, which has obtained the largest number of points cumulated along the path leading from the root to that node.

Both LMAT and Kraken do not use the cumulative distribution of *k*-mers and also they do not exploit the alignment searching. Thus, they can be regarded as the hybrid methods, combining two different strategies—the composition-based and similarity search approach.

### Contribution

In this paper, we present CoMeta—a new fast and accurate algorithm for classification o metagenomes (metagenomic reads). We determine the similarity (termed the match score) between the query read and a group of the reference sequences by counting the number of nucleotides in those *k*-mers, which occur both in the read and in the group. The read is classified to that group, for which the match score is the largest. The group is defined as a set of sequences of specific attribution. Typically, this is one of the taxonomic ranks (e.g., phylum, genus). CoMeta employs an efficient *k*-mer counting and indexing algorithm [55]. Its low memory requirements allows us to create the indexes even at high taxonomy tree levels that embrace large groups of sequences. In this way, after having built the indexes, we can quickly search the tree from the root to the leaves, and find the closest match for a given query read. This classification scheme (i.e., analysis of the taxonomy tree from the top) is in contrast to the existing BLAST-based methods, which require the query read be compared with every reference sequence.

The main idea of the proposed method is similar to the one used in FACS. However, CoMeta does not impose any restrictions on the size of the data. We are able to classify sequences derived from both bacteria and big eukaryotes. The details of our algorithm are given in Section Methods. Extensive experimental study, whose results are reported in Section Results and Discussion, confirms that our algorithm is competitive, offering high speed and accuracy, compared with the state-of-the-art methods.

## Methods

### Introduction

In the following description of our algorithm, several symbols will be used. For clarity, we gathered them in Table 1.

**Table 1:** Dictionary of symbols and acronyms used in the description of the classification.

*ξ*	–	match score, similarity between query read and the set of the reference sequences
Ξ	–	match rate score, percentage ratio of the match score to the read length
*D*_*i*_	–	*k*-mer database for an *i-th* group
*f*	–	number of various groups to which the reads were classified
*F*	–	output files after assignment to the best group
*FP*	–	number of incorrectly classified reads
*G* ^0^	–	set of all reference sequences
$G_{i}^{j}$	–	set of reference sequences for the *i-th* group at the *j-th* level
*k*	–	subsequence (*k*-mer) length
*M*	–	dataset of reads
*MC*	–	match cut-off value of sequence identity
*n* ^*j*^	–	number of various sets (groups) of reference sequences, with whom the query read is compared at the *j-th* taxonomic rank
*NC*	–	number of reads not classified to any group
*R*	–	query read
*S*	–	reference sequence
*TP*	–	number of correctly classified reads

**Fig 1:**
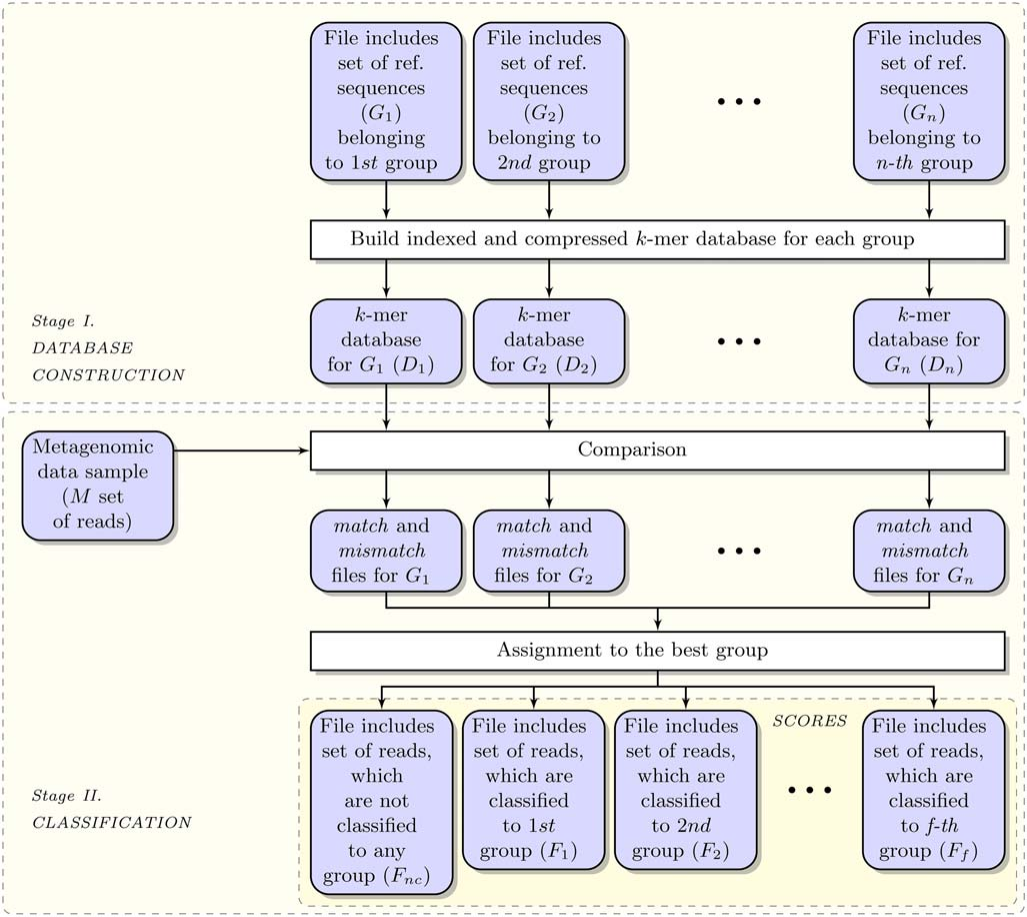
The processing pipeline for metagenomic reads classification for a single rank. In order to avoiding obfuscating the schema, the upper index *j* is not added to the symbols, indicating the *j*-th level of taxonomic classifications.

**Fig 2:**
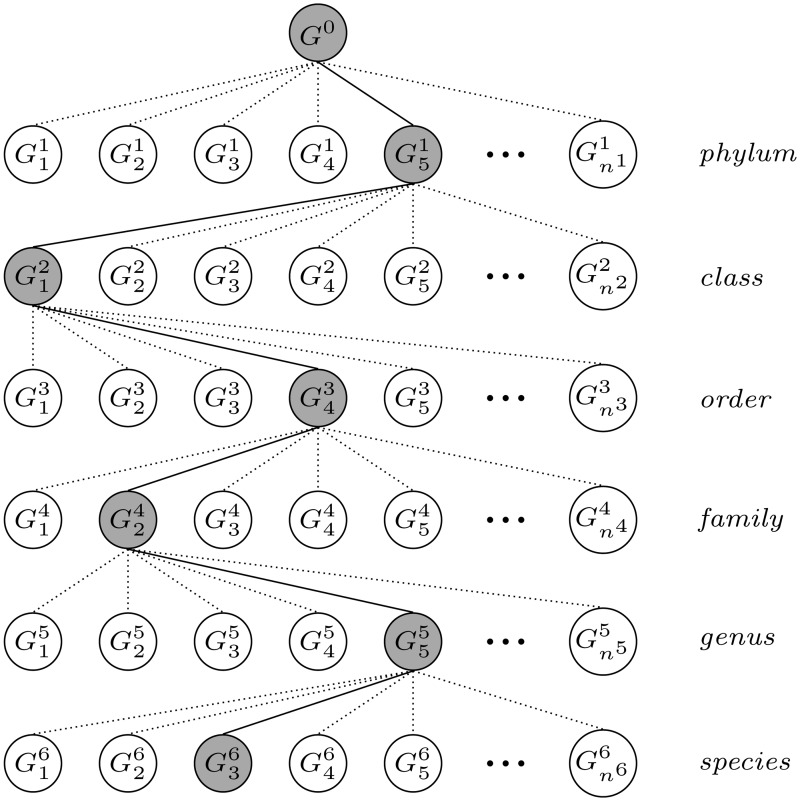
Taxonomy tree-based classification. Iterative execution of stage II (Classification) in Fig 1.

The proposed method consists of two major stages outlined in Figs 1 and 2. Firstly (in the *database construction* stage), the indexed *k*-mer databases of clustered reference sequences are constructed. Subsequently (in the *classification* stage), the reads are classified to various groups with the use of the databases. The second stage is composed of two steps. In the *comparison* step, the input reads are scored according to a number of databases ({*D*_*i*_}). In the *assignment* step, the reads are assigned to the best group. What is important, the classification stage is performed iteratively (for taxonomic classification) to search the taxonomy tree downwards.

The files with the input reads and reference sequences must be given in the FASTA format. The reference sequences and reads contain sometimes the unknown nucleotides (Ns). The *k*-mers with such symbols are skipped.

### Database construction

Before classification, the reference sequences have to be grouped into *n* categories, with whom we want to compare the metagenomic data. For example, the sequences can be grouped according to a phylum, so that a single group contains all the reference sequences belonging to Actinobacteria, Proteobacteria, Thermotogae, etc.

In order to classify the reads into a taxon, the nucleotide sequence database (nt data with entries from all traditional divisions of GenBank, EMBL, and DDBJ) has to be downloaded from the NCBI website. After that, the *tax* number (Taxonomic Identification ID) should be added to each reference sequence using the *gi* number (Sequence Identification ID). The *tax* number is necessary to categorize the sequences into groups. Hence, *gi_taxid_nucl.dmp* file, which contains the links between the *gi* and *tax* number, should also be downloaded from the NCBI website. This file is of a huge size, therefore we created an auxiliary program to avoid loading the entire file into RAM. This program splits the input file into smaller ones, then each of them is read sequentially, and finally the program extracts information about the *tax* number. Detailed support on how to prepare the data is given in readme.txt file in the CoMeta package.

The *k*-mer database *D*_*i*_ for each group *G*_*i*_ is created using a parallel disk-based algorithm, which we derived from our earlier *k*-mer counting software [55]. First, every reference sequence from the group is scanned symbol by symbol to extract all *k*-mers. Subsequently, the *k*-mers are collected and sorted lexicographically. This makes it possible to create the set of all *k*-mers, occurring at least once in the reference sequences (after sorting, the repeating *k*-mers are at adjacent positions, so we can store only a single copy of each one).

The database is stored to the disk in a compact way (compact database). Each nucleotide is encoded using 2 bits. Instead of writing whole *k*-mers to the file, the *k*-mers sharing a common prefix are broken down into two parts, i.e., a four-nucleotide prefix and a suffix, thus, each suffix is saved on 2(*k*−4) bits. The prefix is written once, and it is followed by a list of the suffixes with the number of each occurrences.

For classification purposes, CoMeta uses mainly two lists: 1) a buffer that contains sorted suffixes (stored on 1 byte) after cutting off eight-nucleotide prefix; 2) a list of 65,536 (= 4^8^) elements of information, where the list of suffix for each prefix begins. These lists are built at the beginning of the classification process. However, in order to accelerate the loading of the database during the classification (which is crucial if the same database is used many times), compact database can be converted into a bit larger file (non-compact database), which contains among others the two lists. This file is loaded into the program once, and the size of this file is equal to the size of the memory that the *k*-mer database occupies during the classification.

### Classification

As it was mentioned earlier, the classification of the reads at a single level *j* (e.g., the order) consists of two steps: comparison and assignment. In the following subsections on the taxonomic classification, these steps are described for the *j*-th taxonomic rank. In order to avoid obfuscating the notation, the upper index *j* is omitted.

#### Comparison step

**Fig 3:**
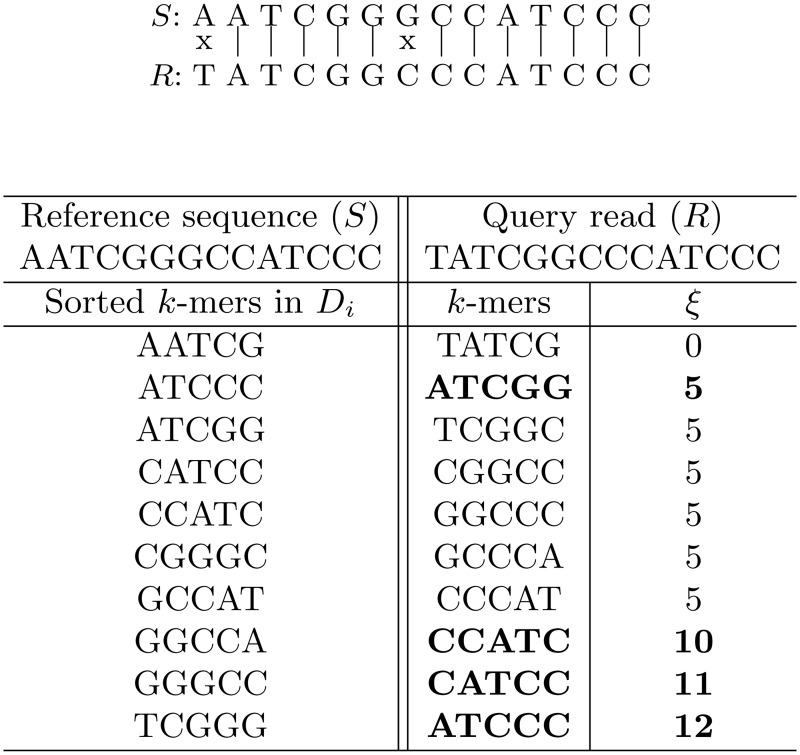
An example of comparing the query read with the reference sequence.

In the comparison step, the set of reads *M* is compared with all *n* *k*-mer databases that have been created beforehand. Each database *D*_*i*_ is loaded into RAM. When comparing each read from *M* against *G*_*i*_ group (1 ≤ *i* ≤ *n*), the match score (*ξ*) is obtained by cumulating the similarity between the *k*-mers extracted from the read and from the reference sequences in *G*_*i*_. For a given read *R*, the successive *k*-mers are obtained using the 1-base sliding window. All possible subsequent *k*-mers from *R* are checked for occurrence in *D*_*i*_. For each *j*-th *k*-mer of *R* found in *D*_*i*_, the match score *ξ* is increased by *ξ*_*j*_, which is the number of bases in the *k*-mer that have not yet contributed to the match score (i.e., $\xi_{j}=k-o_{b}$, where $o_{b}$ is the number of overlapping bases between the *j*-th *k*-mer and the previous *k*-mer found in *D*_*i*_). Due to the 1-base sliding window, two subsequent read *k*-mers have *k*−1 overlapping bases, and our intention is to prevent from increasing the match score too much, if both exist in *D*_*i*_. The number of the overlapping bases between the *p*-th and *q*-th *k*-mers (*p* < *q*) is $o_{b}=\max(k-q+p,0)$. An example on how the match score is calculated is presented in Fig 3 for *k* = 5. For simplicity, we assume that *G*_*i*_ group contains only one reference sequence, *S*. In the “*k*-mers” column, the *k*-mers that occur in the query read are sequentially listed. Those *k*-mers, which are found in *D*_*i*_ database, are marked in bold (a sorted list of the *k*-mers from *G*_*i*_ group is shown in the left part of the figure). The final match score for the sample read is 12, and the match rate score (Ξ), which is percentage ratio of the match score to the read length, is 85.7% (Ξ = 12/14⋅100%). For better illustration, the sequence matching is also shown at the top of the figure.

In order to quickly decide whether a read can obtain a significant score for each group *G*_*i*_, we perform simple filtering. We use the *k*′-base offset sliding window to scan the query read (1 < *k*′ ≤ *k*, for *k*′ = 1 this step is skipped). If none of such *k*-mers exist in *D*_*i*_ we resign from scoring it according to *D*_*i*_. *R* is pre-assigned to the *G*_*i*_ group, if it (or its reverse complement) accumulates a match rate score exceeding a chosen match cut-off value (*MC*). After the comparisons, for each group, we obtain two output files with the preliminary assignments, namely: 1) the *match* file that contains the reads, which accumulated a sufficient match rate score (Ξ ≥ *MC*), and 2) the *mismatch* file which contains the remaining reads. Thus, 2*n* output intermediary assignment files are obtained after the first step of the classification. These files do not contain the nucleotide sequences, but only the single-line description of each read in the FASTA format, along with the obtained match scores. The corresponding nucleotide sequences are added after completing the classification stage.

The idea of this step is similar as used in the FACS algorithm. However, in FACS, the Bloom filters, which are of a limited capacity, are used to store the *k*-mers. For each reference sequence, a separate Bloom filter is created. In addition, long sequences (≳ 200 Mbp) have to be split into a few subsequences, and then Bloom filters are created separately for each of them. Furthermore, usage of the Bloom filters may result in obtaining false *k*-mer positives. In FACS (the Perl implementation), the reads which have been classified as belonging to some reference sequence, are withdrawn from further querying (the sequences are analyzed in some arbitrary order). This approach may result in classifying the read to an incorrect reference, if its match score is over the cut-off value for more than a single reference sequence, but the correct one does not appear as the first one.

#### Assignment to the best group

The second step of the classification stage consists in the analysis of the intermediary assignment files, and the query read is classified to that group, for which the match score (*ξ*) is the highest. When multiple groups obtain the same highest match score, the read could be assigned to: 1) all of these groups; 2) any group; 3) random group.

To increase the sensitiveness of our method, in this step not only *match* but also *mismatch* files can be used. Using the latter, larger percentage of reads are classified, but in some cases this is achieved at the expense of precision. When taking into the account the *mismatch* file, the read is classified to a group with the highest match, even if it is below *MC*. However, this matching must contain at least one matching *k*-mer (*ξ* ≥ *k*).

After this step, the classification is completed for a single taxonomic rank, and *f*+1 output files (*F*) are obtained. Apart from the classified reads, those reads which have not been assigned to any group, are stored in the additional *F*_*nc*_ file. The number *f* is equal to the number of groups, to which the reads from *M* were classified. For the groups without any reads preassigned, the files are not generated at all (hence, *f* ≤ *n*).

For classification to a lower rank, classification stage has to be repeated, which is described in the following subsection.

### Taxonomy tree-based classification

Our taxonomic classification method starts from some high taxonomic rank, and then, if necessary, classifies reads to the lower levels. The search may be started from the superkingdom rank, however, due to very large collections of sequences which contain various groups, we suggest to begin from the phylum.

For the *j*-th taxonomic rank, each read is compared to *n* ^*j*^ groups and it is classified to that group ($G_{b}^{j}$), for which the match score is the highest. Next, the read is compared with those groups at a lower rank (*j*+1), which are subgroups of $G_{b}^{j}$ ($G_{i}^{j+1}\subseteq G_{b}^{j}$, 1 ≤ *i* ≤ *n* ^*j*+1^). Fig 2 shows the taxonomy tree-based classification scheme with an example of the classification path (solid lines). The gray shade indicates a set of the reference sequences, where a query read was classified ($G_{b}^{j}$). In the tree, there are only six basic taxonomic ranks presented, however the process may include other ranks such as subphylum, superclass, etc.

During the classification of the *M* ^*j*^ set of reads (at the *j*-th taxonomic rank), the files $\left\{F_{i}^{j}\right\}$ (*i* = 1,2,…,*f* ^*j*^) are obtained, each of which contains the reads assigned to a particular *i*-th group. In the classification at the next level (*j*+1), the output file from the previous step (*F*_*i*_) is used as the input file, i.e., *M* ^*j*+1^ = *F*_*i*_.

## Results and Discussion

### Implementation and test setup

The algorithms proposed in this paper are implemented in C++ language. The only exception is the tool grouping the reference sequences according to the taxonomic rank, which is implemented in Perl based on Perl module *Bio::LITE::Taxonomy::NCBI* from the *Comprehensive Perl Archive Network*. CoMeta package contains programs for the following tasks:

1. Adding the *tax* number to the single-line description of each reference sequence.
2. Building *k*-mer databases.
3. Two steps of the classification.

The package and documentation are freely available at https://github.com/jkawulok/cometa, all the data used in this paper are available at http://dx.doi.org/10.7910/DVN/29265.

The experiments were conducted on a computer equipped with 12-core Intel Xeon clocked at 2.67 GHz and 96 GB RAM.

CoMeta is a similarity search method, thus we compare it with four other programs from this category. We also examine LMAT and Kraken, which are hybrids of composition-based and similarity search methods also using *k*-mers.

The experiments are divided into two major parts. In the first one, our program was compared to FACS and each read was classified directly to a single reference sequence. This means that each *group* (c.f. Fig 1) contained only one reference sequence (e.g., *group* = ‘Escherichia coli str. K-12 substr. DH10B’) and there was only one level. In the second part of our experiments, the reads were classified to the taxonomic ranks, thus the *level* was taxonomic rank and the *group* was one of the groups at the taxonomic rank, e.g, *level* = ‘phylum’ and *group*=‘proteobacteria’. The classification results for CARMA (command line version 3.0), MEGAN (4.61.5), MG-RAST (3.0), and MetaPhyler (1.13) were taken from Bazinet–Cummings’ paper [56] due to long computation time (in total approximately 34,000 CPU hours). The experiments for LMAT (1.2.1), Kraken (0.10.4b) were made by us.

We assessed the quality of the read classification taking into account the following criteria:

- **Time**: CPU classification time.
- **Memory**: the maximal memory usage during the classification.
- **Classified**: the overall percentage of reads that were classified ($\frac{\text{TP}+\text{FP}}{\text{all}}$), where *TP* and *FP* are numbers of correctly and incorrectly classified reads, respectively.
- **Sensitivity**: the fraction of the correctly classified reads ($\frac{\text{TP}}{\text{all}}$).
- **Precision**: the percentage of correctly classified reads among all classified reads ($\frac{\text{TP}}{\text{TP}+\text{FP}}$).

### Datasets

The experiments were made for the following datasets:

1. *FACS 269 bp*—simulated 454 metagenomic dataset containing 100,000 reads of an average length 269 bp. This dataset was proposed by Stranneheim et al. [49] and we downloaded it from FACS website. The reads are from 17 bacterial genomes (four various phyla rank), three archaeal genomes (two various phyla rank), three viral genomes, and two human chromosomes. After removing reads containing more than 50% of unknown nucleotides, dataset of 93,653 reads was obtained, which we called *reduced FACS 269 bp*.
2. *MetaPhyler 300 bp*—simulated metagenomic dataset containing 73,086 reads of length 300 bp. This dataset, proposed by Liu et al. [47], was obtained from 31 phylogenetic marker. Unfortunately, some reads had no information about their origin and it would be impossible to verify whether they were correctly classified or not, so we filtered them out. Finally, 66,841 reads were left and used for our experiments. The reads have been derived from the organisms belonging to 17 various phyla. The majority originate from Proteobacteria (51%) and Firmicutes (21%).
3. *CARMA 265 bp*—simulated 454 metagenomic dataset containing 25,000 reads of an average length 265 bp. This dataset was proposed by Gerlach and Stoye [45]. We downloaded it from WebCARMA website. The distribution of the reads in the bacterial phyla is: Proteobacteria—73.02%; Firmicutes—12.92%; Cyanobacteria—7.83%; Actinobacteria—5.22%; Chlamydiae—1.01%.
4. *PhyloPythia 961 bp*—dataset containing 124,941 random reads of an average length 961 bp from 113 isolate microbial genomes, proposed by Patil et al. [37]. Some reads are repeated in this dataset and only 114,457 reads are unique. The majority of them (81%) come from Proteobacteria. These reads were classified to the genus rank (Rhodopseudomonas—21.00%; Bradyrhizobium—20.06%; Xylella—9.16%; the rest—each one below 6%).
5. *HiSeq 92 bp*—dataset containing 10,000 reads of an average length 92 bp, proposed by Wood and Salzberg [51]. It was built using 20 sets of bacterial whole-genome shotgun reads and generated by Illumina HiSeq sequencing platform.
6. *MiSeq 156 bp*—dataset containing 10,000 reads of an average length 156 bp, proposed by Wood and Salzberg [51]. It was built using 20 sets of bacterial whole-genome shotgun reads and generated by Illumina MiSeq sequencing platform.

The 2nd–6th datasets contain reads from bacterial genomes only. Both *FACS 269 bp* and *reduced FACS 269 bp* datasets contain also reads from human, viral, and archaeal species.

### Experiment One

In the first experiment, we compared CoMeta with FACS 2.1 algorithm implemented in Perl [49], and with FACS implemented in C. We tried to reproduce the results reported by Stranneheim et al. [49] (FACS in Perl). Unfortunately, we obtained different scores, despite using their scripts, the same set of parameters, and the same set of 25 reference sequences.

Stranneheim et al. verified false positives using MEGABLAST for *k*-mer length equal to 17, 21, 25, and 35. To speed up this process we constructed a homologous map for comparing reads to the reference sequences. Assuming the same criteria as in FACS, if a read obtains 500 hits with E-values < 10^−50^ using MegaBLAST, then it is considered as a homologue. In this way, the classification results can be quickly checked for large sets of false positives, such as those created for short *k*-mers. The resulting map contained 17 homologous.

As discussed in the previous section, *FACS 269 bp* dataset includes many reads, which consist mostly of unknown nucleotides. Therefore, in order to provide a fair comparison, we removed them and used *reduced FACS 269 bp* dataset. The comparison was performed using the following variants of FACS and CoMeta:

1. *FACS-P*: FACS 2.1 algorithm in Perl. The probability of false positive parameter (*p*_*f*_) in Bloom filter (used by FACS) was set to 0.0005.
2. *FACS-C*: FACS algorithm in C, whose sources were downloaded on 5th February 2014, from https://github.com/SciLifeLab/facs. The reads are classified to each reference sequence to which similarity is highest than *MC*. The probability of false positive parameter in Bloom filter was set to the same value as for *FACS-P*.
3. *pre-CoMeta*: The only comparison step of CoMeta algorithm (without assignment). This is a similar strategy as implemented in *FACS-C*.
4. *CoMeta*: The complete proposed classification algorithm of a read (to all reference sequences) using the best solution (presented in Fig 1).

*FACS-P*, *FACS-C*, and *pre-CoMeta* were ran using various values of *k* and *MC*. In *CoMeta*, we used *MC* = 30% in the “comparison” step, and then the reads were classified to the reference sequence according to the highest score. When *FACS-P* classifies a read to some *G*_*i*_-th reference sequence it does not compare the read with any further reference sequence (*G*_*i*+*j*_, *j* > 0). Since in *FACS-C* and *pre-CoMeta* the reads are compared with each reference sequence, their FP values can be larger than for *FACS-P*.

In Table 2, we report the best classification results obtained using the four aforementioned methods. The results for *CoMeta* are when taking into account the *mismatch* files. When we stopped the algorithm after the “comparison” step (*pre-CoMeta*), the sensitivity was the highest, unfortunately, at the expense of a large number of false positives. *pre-CoMeta* gave slightly better precision score than *FACS-C*. The precision is high for *FACS-P*, however the sensitivity is the lowest here. In general, the best results was obtained by *CoMeta* which was able to classify almost every read and the number of false positives was small.

**Table 2:** Comparison of FACS algorithms with CoMeta.

*k*	*MC*	Sensitivity	Precision	Classified	*t*
	[%]	[%]	[%]	[%]	[hh:mm:ss]
*FACS-P*					
18	80	97.62	97.86	99.76	00:03:14
21	65	97.86	98.08	99.78	00:02:49
21	70	97.82	98.27	99.55	00:02:49
24	55	97.77	98.12	99.64	00:02:36
27	45	97.65	98.07	99.58	00:02:27
*FACS-C*					
17	30	**99.92**	90.20	99.93	00:01:08
17	40	98.78	93.25	98.78	00:01:12
19	30	99.48	92.65	99.48	**00:00:49**
21	30	98.26	94.27	98.27	**00:00:43**
*pre-CoMeta*					
15	55	99.30	93.56	99.31	00:01:52
18	45	99.42	93.36	99.43	00:01:21
21	45	99.05	93.93	99.06	00:01:08
25	30	99.56	92.05	99.57	00:01:09
27	35	99.36	93.07	99.37	00:01:16
*CoMeta*					
18	–	97.91	97.91	**100.00**	00:01:37
21	–	98.40	98.41	99.99	00:01:36
24	–	98.69	98.75	99.93	00:01:37
27	–	98.71	**99.08**	99.63	00:01:30

Comparison of the best classification results obtained using four methods (bold values indicate the best score for each column):

*FACS-P*: the FACS 2.1 program in Perl [49]. When read is classified to some *G*_*i*_-th reference sequence, it does not be compared with any further reference sequence;

*FACS-C*: the FACS program in C, which was downloaded from https://github.com/SciLifeLab/facs. The reads are classified to each reference sequence to which similarity is highest than *MC*;

*pre-CoMeta*: the only comparison step of CoMeta algorithm (without assignment). This is a similar strategy as implemented in *FACS-C*.

*CoMeta*: the full proposed algorithm, the reads are classified to the reference sequence according to the highest score.

**Fig 4:**
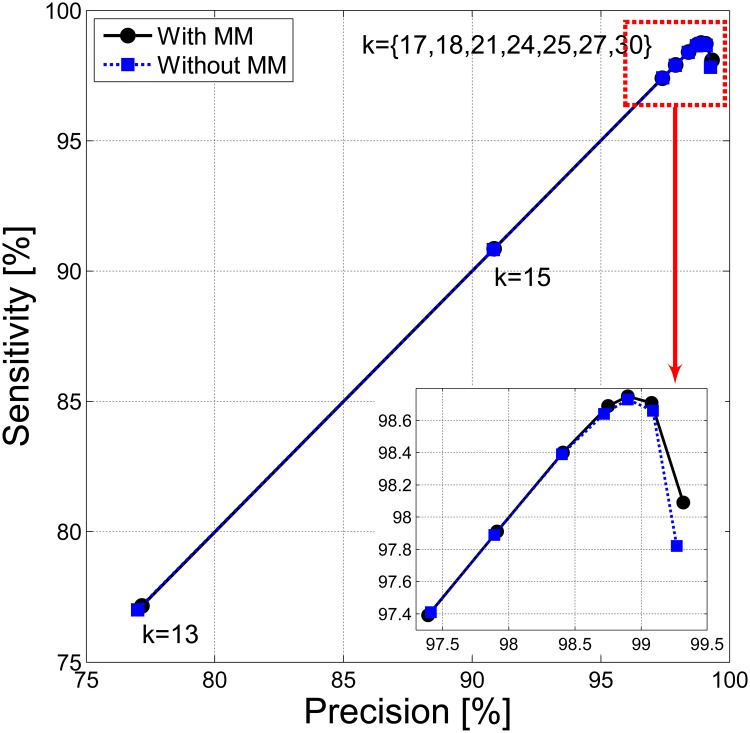
Classification accuracy for *CoMeta* in Experiment One. Accuracy of classification is shown when taking into account only the *match* files (dotted line with square mark) and when considering additionally the *mismatch* files (solid line with a circle mark). The performance curve reflects various *k*-mer lengths.

The precisions and sensitivities for *CoMeta*, depending on *k*, are shown in Fig 4. The results are presented with and without taking into account the *mismatch* files (*MM*). It may be noticed that for growing *k* up to *k* = 25 both precision and sensitivity grows, then sensitivity falls down. The reason is that with the increase of *k*, the number of unclassified sequences also increases.

**Fig 5:**
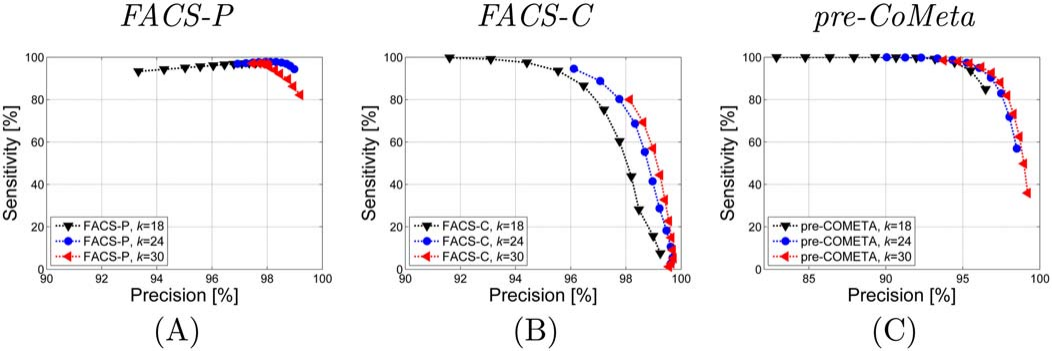
Classification accuracy for the Experiment One using various *k* parameter. The plot A represents scores after classification using *FACS-P*, the plot B—using *FACS-C*, and the plot C—using *pre-CoMeta*. Each series shows the results for 11 different threshold values, in sequence starting from the left part of each figure: *MC* = 30,35,40,…,80 [%].

The sensitivity and precision for *FACS-P*, *FACS-C*, and *pre-CoMeta* for various *k* are presented in Fig 5A–5C. Each series shows the results for 11 different threshold values, in sequence starting from the left part of each figure: *MC* = 30,35,40,…,80 [%]. It can be seen from the plot A that only for a small value of *k* in *FACS-P*, the sensitivity does not drop with the increasing threshold values, while in other cases, the sensitivity for a large *MC* declines. The detailed analysis of the impact of the parameters *k*, *MC* and *p*_*f*_ (for building the Bloom filters) on the accuracy of *FACS-P* was presented in our earlier study [57].

The processing times of the examined methods are given in Table 2. It can be seen that *FACS-C* is usually the fastest, however, *CoMeta* is slower only by a factor two.

### Experiment Two

The second experiment consisted in classifying reads to the taxonomic groups. We compared our method with all the examined programs except for FACS.

The programs were evaluated for the 1st–4th metagenomic datasets (from the 454 sequencing). As was said the results for CARMA, MEGA, MG-RAST, and MetaPhyler were taken directly from Bazinet–Cummings’ paper [56]. Bazinet and Cummings classified *PhyloPythia 961 bp* at the genus rank, *FACS 269 bp* at the superkingdom rank, and the other two datasets at the phylum rank. When running CoMeta, Kraken, and LMAT we also conducted *PhyloPythia 961 bp* classification into the genus but the three other datasets into the phyla rank.

For the Iluimina datasets (*HiSeq 92 bp* and *MiSeq 156 bp*) we examined Kraken, LMAT, and CoMeta. The classification level was set to the genus rank here.

LMAT was tested for two databases downloaded from the LMAT website: “full” *k*-mer/taxonomy database (*kFull*) and smaller database built from “marker library” (*kML*). These databases were constructed from the complete and partial microbial genome sequences from the NCBI genome database from 2011. The *kFull* database contains 20-mers, while *kML* — 18-mers.

Kraken was evaluated using MiniKraken database (the only available) downloaded from the Kraken website. Unfortunately, Kraken failed to construct the database from our set of reference sequences (probably due to huge memory requirements of Jellyfish tool used to collect *k*-mer statistics). We were also not able to obtain the larger databases from the authors.

For CoMeta, we built *k*-mer databases using all reference sequences from the NCBI genome database from 2012. We divided the sequences into several groups, so during classification we could easily select the groups we wanted to classify to. Therefore, in some experiments we used all sequences (*allDb* database), while in the rest only those from bacteria, viruses, and archaea (*micDb* database). The databases were constructed using various *k*-mer lengths (15, 18, 21, 24, 27, and 30).

We conducted a large number of preliminary experiments for different parameters. Some of them are described in S1 Supporting Information. The most important results of our experiments are summarized in Tables 3 and 4. LMAT results are for “minimum score” (*ms*) set to 0 (optimal value according to the preliminary experiments). The results for CoMeta *allDb* were calculated in such a way that if a read was classified to several groups, then it was assigned to all of them. Hence, in some cases, the sum of TP, FP, and NC was higher than the number of all reads in the dataset. For better comparison of CoMeta and Kraken, the results for CoMeta *micDb* were computed using the same strategy as in Kraken, so if a read was classified to multiple groups we did not assign it to any group.

**Table 3:** Comparison of programs using 454 reads.

Program	FACS 269bp	MetaPhyler 300bp	CARMA 265bp	PhyloPythia 961bp
Percentage of classified reads				
CARMA^a^	29.0	93.6	68.7	61.3
MEGAN^a^	48.4	88.2	90.5	62.2
MetaPhyler^a^	0.2	80.9	0.5	0.6
MG-RAST^a^	27.1	29.8	80.2	70.5
LMAT *kML*	24.7(26.4^b^)	96.5	80.4	98.3
LMAT *kFull*	92.5(98.8^b^)	99.3	86.0	82.7
MiniKraken	—	100.0	96.7	98.0
CoMeta *allDb*	93.6(100.0^b^)	100.0	99.9	94.7
CoMeta *micDb*	—	100.0	98.9	97.4
Sensitivity (percentage)				
CARMA^a^	26.7	93.4	68.5	59.8
MEGAN^a^	42.5	87.9	90.3	61.0
MetaPhyler^a^	0.1	80.7	0.5	0.5
MG-RAST^a^	25.0	29.7	80.1	67.2
LMAT *kML*	24.7(26.3^b^)	95.7	80.4	98.1
LMAT *kFull*	92.5(98.7^b^)	98.5	86.0	82.5
MiniKraken	—	99.9	96.7	97.7
CoMeta *allDb*	93.4(99.7^b^)	99.6	99.1	94.1
CoMeta *micDb*	—	99.8	98.9	96.2
Precision (percentage)				
CARMA^a^	92.0	99.7	99.7	97.4
MEGAN^a^	78.1	99.7	99.8	98.1
MetaPhyler^a^	84.0	99.7	100.0	83.8
MG-RAST^a^	92.4	99.8	99.9	95.3
LMAT *kML*	99.9(99.9^b^)	97.8	100.0	99.8
LMAT *kFull*	100.0(100.0^b^)	97.8	100.0	99.8
MiniKraken	—	99.9	100.0	99.7
CoMeta *allDb*	99.8(99.8^b^)	99.6	99.1	99.3
CoMeta *micDb*	—	99.8	99.9	98.8

^a^—The results of the program are taken from the Bazinet–Cummings’ paper [56].

^b^—The results for *FACS 269bp* dataset, where reads with more than 50% of unknown nucleotides (Ns) are filtered out. The values outside the brackets are for the whole dataset.

CoMeta *allDb* parameters: *MC* = 30%, *k* = 24.

CoMeta *micDb* parameters: *MC* = 5%, *k* = 30.

LMAT *kML* and *kFull* parameter: *ms* = 0.

**Table 4:** Comparison of programs for various level classification using Illumina reads.

Programs	HiSeq 92 bp			MiSeq 156 bp		
	Sensitivity	Precision	Classified	Sensitivity	Precision	Classified
PHYLUM						
LMAT *kFull*	89.89	99.74	90.12	88.23	99.47	88.70
MiniKraken^a^	65.34	99.79	65.48	75.88	99.93	75.93
CoMeta *micDb*	81.64	98.97	82.49	86.71	99.11	87.49
CLASS						
LMAT *kFull*	88.06	99.66	88.36	85.79	99.65	86.09
MiniKraken^a^	65.16	99.65	65.39	75.73	99.91	75.80
CoMeta *micDb*	80.87	98.14	82.40	86.34	98.83	87.36
ORDER						
LMAT *kFull*	86.48	99.80	86.65	81.00	99.63	81.30
MiniKraken^a^	64.89	99.51	65.21	75.52	99.87	75.62
CoMeta *micDb*	80.34	97.73	82.21	85.39	98.01	87.12
FAMILY						
LMAT *kFull*	84.96	99.79	85.14	79.40	99.72	79.62
MiniKraken^a^	64.75	99.46	65.10	75.43	99.81	75.57
CoMeta *micDb*	80.13	97.61	82.09	85.05	97.76	87.00
GENUS						
LMAT *kFull*	84.74	99.80	84.91	73.75	99.53	74.10
MiniKraken^a^	64.54	99.45	64.90	71.95	98.04	73.39
MiniKraken^b^	66.12	99.44	—	67.95	97.41	—
Kraken^b^	77.15	99.20	—	73.46	94.71	—
Kraken-GB^b^	93.75	99.51	—	86.23	98.48	—
CoMeta *micDb*	79.82	97.44	81.92	77.50	90.83	85.32

^a^—The results of the program are counted by ourselves.

^b^—The results of the program are taken from the Wood–Salzberg’ paper [51].

CoMeta *micDb* parameters: *MC* = 5%, *k*=24. LMAT *kFull* parameter: *ms* = 0.

In both variants of CoMeta (*allDb* and *micDb*), the *mismatch* files were taken into account, when the reads were being assigned to the best groups. Depending on the dataset and database, the best classification results were obtained for different values of *k*. Using *micDb*, the best accuracy for the Illumina reads (which are short) was obtained using shorter *k*-mers (i.e., *k* ≈ 24). For long reads (after the 454 sequencing) the most accurate classification scores were obtained for *k* ≈ 30. However, using *allDb*, where reads were assigned to many groups, the best classification results were obtained for *k* = 24.

The difference in the number of reads between the *reduced FACS 269 bp* and the original dataset is 6,347 (these are the reads containing more than 50% of unknown nucleotides). Differences in the classification results for the original and *reduced FACS 269 bp* datasets using CoMeta and LMAT were in the number of unclassified reads and equal 6,346 and 6,347 reads, respectively. Obviously, real reads may contain unknown nucleotides, however in our opinion during the validation of the classifiers, ambiguous reads should not be treated equally, as the reads of all known nucleotides. Therefore, the classification results in Table 3 (using CoMeta and LMAT) are given both for the *FACS 269 bp* and the *reduced FACS 269 bp* datasets.

The greatest differences in the classification results between the tested programs were observed for the *FACS 269 bp* dataset, which includes 72,951 reads derived from a human chromosome. CoMeta *allDb* and LMAT *kML* classified the majority of reads, significantly outperforming other programs. The databases used by MetaPhyler, MG-RAST, LMAT *kML*, as well as CoMeta *micDb* do not contain human sequences, or contain only specific marker genes, so it is understandable that the results are rather poor. Although the databases in CARMA and MEGAN contain human sequences, the results obtained on these metagenomic datasets were also poor. To investigate this problem, we tried to align a few reads from this dataset using BLASTX (both programs employ it), and BLASTX failed to classify some reads, which explains weak results for CARMA and MEGAN. LMAT *kML* classified incorrectly fewer reads than CoMeta *micDb*, but also fewer reads were classified correctly, hence the total number of classified reads was smaller for LMAT than for CoMeta.

For three other datasets, the results of MetaPhyler, MG-RAST, CARMA, and MEGAN were better than those achieved for *FACS 269 bp*, however, LMAT, CoMeta, and Kraken were able to classify more reads. MetaPhyler is very fast since it uses only the “marker genes”, however only reads having them are correctly classified. Thus, this algorithm performs well only for the dataset created by the program’s authors. During DNA sequencing, only a certain percentage of reads have the marker genes, therefore in many cases MetaPhyler does not recognize correctly the origin of the reads. The best results for the *MetaPhyler 300 bp* dataset were obtained by Kraken and CoMeta, which outperformed LMAT. For the *CARMA 265 bp* dataset the winner was CoMeta. Kraken returned slightly worse scores, and LMAT—much worse. However, for the *PhyloPythia 961 bp* dataset, it was LMAT *kML*, which achieved the best score. Nevertheless, it is worth noting that the results of LMAT *kFull* was significantly worse (comparing only those three programs), whereas for the remaining datasets the classification results were better using *kFull* than using *kML* database.

Table 4 summarizes the evaluation of CoMeta, LMAT, and Kraken for the Illumina reads. Here we showed results for five classification levels: phylum, class, order, family, and genus. As mentioned earlier, we run Kraken using only the MiniKraken database downloaded from the Kraken website, because we have not managed to build the larger database nor to obtain it Kraken’s authors. Therefore, in addition to the results obtained in our experiment, we present also the results quoted from Wood–Salzberg’ paper [51] (that work reports the results only for the genus level). Although we carefully followed the instructions when running Kraken, we obtained different results for two datasets using MiniKraken database, compared with those reported in [51]. The precision values were similar, but the difference in sensitivity was greater. For the *HiSeq 92 bp* dataset, we obtained the sensitivity 1.58% smaller than reported in [51], and for the *MiSeq 156 bp* dataset it was 4% higher. The differences in precision could be due to the fact that Kraken’s authors took into account the reads incorrectly classified to the levels above the analyzed rank, whereas we consider such reads unclassified. However, we cannot explain the cause of the difference in the sensitivity values. The best classification results for both datasets at the genus level were obtained using Kraken-GB. This database, according to its authors, contains GenBanks draft and completed genomes for bacteria and archaea. Taking into account the results obtained in our experiments, the *HiSeq 92 bp* dataset was classified the best by LMAT and by CoMeta. For the *MiSeq 156 bp* dataset, LMAT was better than CoMeta only at the phylum level, while CoMeta correctly classified much more reads at lower levels.

In Table 5 we present the classification times and memory usage. It may be seen that the programs which use *k*-mers databases use a lot of memory. Using all available reference sequences (*allDb*), CoMeta consumed about 70 GB of RAM. This was reduced to 20 GB, when taking into account only bacteria, viruses, and archaea (*micDb*). CoMeta *allDb* is by 1.5–2 times slower than CoMeta *micDb*. MiniKraken database contains only a fraction of *k*-mers of the reference sequence complete genomes for bacteria, viruses, and archaea; it consumed between 1.5 GB and 4 GB of RAM. When using the complete database without eukaryotes Kraken needs 74 GB (according to the authors).

**Table 5:** Comparison of RAM memory usage and CPU times.

Program	FACS	MetaPhyler	CARMA	PhyloPythia	HiSeq	MiSeq
	269bp	300bp	265bp	961bp	92bp	156bp
CPU Runtime (minutes)						
CARMA^a^	290880	77340	74950	360107	—	—
MEGAN^a^	288020	72060	72010	351060	—	—
MetaPhyler^a^	10	20	2	28	—	—
MG-RAST^a^	60	10080	20160	12960	—	—
LMAT *kML*	36(60^b^)	58	43	348	—	—
LMAT *kFull*	54(93^b^)	213	38	772	15	33
MiniKraken	—	1.22	1.07	2.95	1.3	1.2
CoMeta *allDb*	41(76^b^)	14	28	144	—	—
CoMeta *micDb* (ph)	—	9	14	35	8	9
CoMeta *micDb* (ge)	—	—	—	79	42	68
Memory Usage (Megabytes of RAM)						
CARMA^a^	100	100	100	120	—	—
MEGAN^a^	1024	1024	1024	1410	—	—
MetaPhyler^a^	5734	5734	5734	5734	—	—
MG-RAST^a^	—	—	—	—	—	—
LMAT *kML*	17000(17284^b^)	17019	2128	13311	—	—
LMAT *kFull*	9295(9481^b^)	13247	13286	15092	5807	12392
MiniKraken	—	4098	3210	4100	1317	1449
CoMeta *allDb*	71260(71903^b^)	70743	71313	69508	—	—
CoMeta *micDb*	—	19552	19320	19552	10297	17689

^a^—The results of the program are taken from the Bazinet–Cummings’ paper [56].

^b^—The results for *FACS 269bp* dataset, where reads with more than 50% of unknown nucleotides (Ns) are filtered out. The values outside the brackets are for the whole dataset.

*FACS 269 bp*, *MetaPhyler 300 bp*, and *CARMA 265 bp* datasets were classified to phylum level, whilst *PhyloPythia 961 bp*, *HiSeq 92 bp*, and *MiSeq 156 bp* datasets to genus level. In the table besides the times of classification to the genus level for CoMeta *micDb* (ge), the times of classification to earlier levels are shown—the phylum levels (ph).

The running time of CoMeta *micDb* when classifying to the genus level for the *PhyloPythia 961 bp* dataset, compared with the *HiSeq 92 bp* dataset, was only twice longer, although both the number of reads and their lengths are about ten times larger (hence, the file size is over 100 times larger). This results from the fact that loading the *k*-mer database takes much more time than classification of the reads. Kraken is the fastest among the examined programs. Compared to LMAT, CoMeta was faster when classifying to the phylum level. For classification to the genus level, CoMeta was faster only for a big dataset (*PhyloPythia 961 bp*), while the small datasets with short reads (*HiSeq 92 bp* and *MiSeq 156 bp*) were classified faster by LMAT.

### Databases building

The *k*-mer/taxonomy databases consist of all reference sequences downloaded from the NCBI website. As it has been discussed earlier, we suggest the read classification be started from the phylum rank. The “raw” genome database used in this study was downloaded on July 2012. The 13 nt files included: 261,295 sequences from Archaea, 4,036,205 from Bacteria, 10,205,401 from Eukaryota, 3,127 from Viroids, and 1,175,053 from Viruses. Apart from 15,681,081 sequences of a known origin and defined superkingdom, 509,677 sequences were undefined (for example plasmids, artificial sequences, or environmental samples).

Each sequence had Sequence Identification ID (*gi*), which was used to set Taxonomic Identification ID (*tax*). The sequences were divided into groups according to the rank of phylum, plus for group Viruses and Viroids. Overall, 99 groups were established (c.f. Table 6, row “num groups”).

**Table 6:** Compact *k*-mer database, where the reads are classified into the phylum rank.

	Archaea	Bacteria	Eukaryota	Viroids	Viruses	Total
num groups	6	36	55	1	1	99
num seq	261,295	4,036,205	10,205,401	3,127	1,175,053	15,681,081
*k* = 15	1.9 GB	17.0 GB	29.9 GB	1.1 MB	1.1 GB	49.9 GB
*k* = 18	2.2 GB	34.4 GB	93.7 GB	1.1 MB	1.4 GB	131.7 GB
*k* = 21	2.3 GB	37.6 GB	111.9 GB	1.2 MB	1.5 GB	153.3 GB
*k* = 24	2.3 GB	39.0 GB	117.4 GB	1.3 MB	1.6 GB	160.4 GB
*k* = 27	2.4 GB	39.3 GB	120.9 GB	1.4 MB	1.7 GB	164.2 GB
*k* = 30	2.4 GB	39.6 GB	123.3 GB	1.4 MB	1.8 GB	167.0 GB

The total size of the compact *k*-mer databases for groups of the phylum rank at various lengths of *k*-mer. The number of groups belonging to the superkingdom is given in the first row, and the number of the sequences is in the second one. The sizes of each dataset are provided in S1 Supporting Information.

In the reported experiments, we divided the sequences into overlapping *k*-mers of different lengths, *k* = 15,18,21,24,27,30, hence, we obtained six different database setups. In order to accelerate loading of the database during classification, we used non-compact databases. The overall sizes of the databases for classification at the phylum rank are presented in Table 6, with the number of groups belonging to the superkingdom. Sizes for all non-compact databases that are loaded into RAM during the “Comparison” step (c.f. Fig 1), are provided in S1 Supporting Information. The largest *k*-mer database is for the “Chordata” phylum (up to 73 GB for *k* = 30), however in many metagenomic studies, the eukaryotes are not investigated at all. For bacteria, the Proteobacteria *k*-mer database is the largest one (almost 20 GB of RAM is necessary).

The dependence of the database size on the number of unique *k*-mers (which appeared at least once in the *G*_*i*_ group) is shown in S1 Supporting Information. Approximately, the relationship between *k* and the database size is linear. The size of the non-compact database is approximately equal to the compact one for *k* = 30.

## Conclusions and future work

In this paper, we proposed a new method for classification of reads to the taxonomic rank. First, the groups of reference sequences (each derived from a single taxon) are divided into overlapping *k*-mers (short substrings), from which the databases are built. Each database is subsequently used for checking the similarity between the query read and the group, which this database represents. We proceed the read classification from the root towards the leaves of the taxonomical tree, which accelerates the program execution, since the read does not have to be compared with each reference sequence. The presented experimental results proved our approach to be competitive and outperforming many alternative popular programs. The results also indicate how important it is to properly select the length of *k*-mers. For too small *k*’s, too many reads are misclassified, while too large *k*’s increase the number of unclassified reads. The downside of our method is that it needs a lot of RAM, when large *k*-mer databases are used. For classification at the phylum level, using the largest set of *k*-mers for Proteobacteria, about 20 GB are required. CoMeta is slower than the very recently published Kraken program. However, CoMeta returns information about all the groups to which the query read was classified if it was classified to several ones, (when the conflict occurred), and not like Kraken and LCAT which cut off the branch and classify the read to a higher level.

Our ongoing research includes examining the influence of the length of the reference sequences (derived from one group) on the best value of the *k* parameter, so that it can be selected automatically. Furthermore, we intend to take into consideration not only the number of matched nucleotides (match scores), but also the number of deletions and insertions.

## Supporting Information

S1 Supporting Information Additional tables and figures of the experiments results. (PDF)
