# Supplementary material for: CoMeta: Classification of Metagenomes Using k-mers
Source: PLoS One. 2015 Apr 17;10(4):e0121453. doi: 10.1371/journal.pone.0121453 (PMC4401624; doi:10.1371/journal.pone.0121453)
Supplement: S1 Supporting Information — (PDF) [file pone.0121453.s001.pdf]

Supplementary Material
CoMeta: Classification of metagenomes using k-mer

Jolanta Kawulok*, Sebastian Deorowicz

Institute of Informatics, Silesian University of Technology, Gliwice, Poland
* E-mail: jolanta.kawulok@polsl.pl

1 Experiment Two

This section includes the presentation of the certain additional results of the classification 454 reads using CoMeta *allDb* and LMAT algorithms.

1.1 CoMeta *allDb*

The *allDb* database was constructed using all reference sequences from the NCBI genome database from 2012, which was mentioned in the main paper. The program with this database was evaluated for *FACS 269 bp*, *Reduced FACS 269 bp*, *MetaPhyler 300 bp*, *CARMA 265 bp*, and *PhyloPythia 961 bp* metagenomic datasets (from the 454 sequencing). *PhyloPythia 961 bp* was classified into the genus, whilst the other datasets into the phyla rank. The classification results were calculated in such a way that if a read was classified to several groups, then it was assigned to all of them. Hence, in some cases, the sum of TP, FP, and NC was higher than the number of all reads in the dataset.

Tables 1 to 5 show the classification results using different parameters and metagenomic sets. For each k-mer length, classification was performed with two options: 1) without taking into account the *mismatch* files (the upper row); 2) with taking into account the *mismatch* files—the reads accumulated the alignment points more than 0 and less than 30% of its length (the bottom row). In most cases, with the *mismatch* files included, the number of true positives increases, and thus the sensitivity increases as well. However, this is achieved at the expense of the accuracy—the number of false classified read increases, simultaneously with the number of the sequences that were classified at all. The length of k-mers, for which the classification results are the best, is not constant and depends on the set of reads. For *CARMA* and *reduced FACS* it is 21, *original FACS* obtained the best score for $k = 24$, while for *PhyloPythia* and *MetaPhyler* the optimal k is 27. However, the differences between the results are not large in the range of $k \in [21, 27]$ for all sets. With the increase of the k-mer length, also the size of the database grows, therefore more memory is needed.

The *PhyloPythia 961 bp* set was classified to the genus rank. However, the classification began from the phylum rank, and then the reads were classified to the class, order, family, and ended up to the genus rank. Accumulation of time, during the classification to a lower rank is shown in Figure 4. As expected with a classification to a lower rank, the number of classified sequences decreased (NC increased), what can be noted in Figure 3, as well as the value of sensitivity and precision decreased (see Figures 1 and 2). The biggest change in sensitivity and precision are noticeable for k-mers shorter than 18 nucleotides, for longer ones the quality of classification did not much decrease, which is due to lower error probability assignment to the rank.

Table 1. Classification results for the original FACS simHC metagenomic data set (100 000 reads, 269 bp) obtained using CoMeta *allDb*

k	TP	FP	NC	Sensitivity [%]	Precision [%]	Classified [%]	t_{com} [hh:mm:ss]	t_{clas} [hh:mm:ss]	t_{all} [hh:mm:ss]	Memory [MB]
15	82241	12263	6346	81.55	87.02	93.65	00:28:36	00:00:26	00:29:02	5497
	82241	12263	6346	81.55	87.02	93.65	00:28:36	00:01:18	00:29:54	5497
18	90098	3684	6346	89.98	96.07	93.65	01:16:19	00:00:09	01:16:28	50479
	90098	3684	6346	89.98	96.07	93.65	01:16:19	00:01:06	01:17:25	50479
21	93532	242	6352	93.41	99.74	93.65	00:35:04	00:00:06	00:35:10	68103
	93532	242	6352	93.41	99.74	93.65	00:35:04	00:01:11	00:36:15	68103
**24**	93554	215	6406	93.39	99.77	93.59	00:40:04	00:00:05	00:40:09	71260
	**93570**	**215**	**6390**	**93.41**	**99.77**	**93.61**	00:40:04	00:00:52	00:40:56	71260
27	93306	217	6659	93.14	99.77	93.34	00:34:55	00:00:06	00:35:01	72175
	93380	227	6585	93.20	99.76	93.42	00:34:55	00:00:49	00:35:44	72175
30	92547	221	7413	92.38	99.76	92.59	00:23:55	00:00:06	00:24:01	75528
	92770	232	7190	92.59	99.75	92.81	00:23:55	00:00:46	00:24:41	75528

The first row is for the classification without taking into account the *mismatch* file, the second row takes it in into account. t_{com} – time of comparing all the reads with all the groups; t_{clas} – time of classifying the reads to the best group. Bold values indicate the best score.

Table 2. Classification results for the reduced FACS simHC metagenomic data set (93 653 reads, 269 bp) obtained using CoMeta *allDb*

k	TP	FP	NC	Sensitivity [%]	Precision [%]	Classified [%]	t_{com} [hh:mm:ss]	t_{clas} [hh:mm:ss]	t_{all} [hh:mm:ss]	Memory [MB]
15	82240	12263	0	87.02	87.02	100.00	00:29:35	00:00:23	00:29:58	6769
	82240	12263	0	87.02	87.02	100.00	00:29:35	00:01:25	00:31:00	6769
18	90097	3684	0	96.07	96.07	100.00	00:40:56	00:00:08	00:41:04	49056
	90097	3684	0	96.07	96.07	100.00	00:40:56	00:01:32	00:42:28	49056
**21**	93531	242	6	99.74	99.74	99.99	00:44:30	00:00:05	00:44:35	68097
	**93531**	**242**	**6**	**99.74**	**99.74**	**99.99**	00:44:30	00:00:58	00:45:28	68097
24	93553	215	60	99.71	99.77	99.94	01:14:29	00:00:04	01:14:33	71903
	93569	215	44	99.72	99.77	99.95	01:14:29	00:00:54	01:15:23	71903
27	93305	217	313	99.44	99.77	99.67	01:21:37	00:00:03	01:21:40	73802
	93379	227	239	99.50	99.76	99.74	01:21:37	00:00:48	01:22:25	73802
30	92546	221	1067	98.63	99.76	98.86	01:49:36	00:00:04	01:49:40	76328
	92769	232	844	98.85	99.75	99.10	01:49:36	00:00:43	01:50:19	76328

The first row is for the classification without taking into account the *mismatch* file, the second row takes it in into account. t_{com} – time of comparing all the reads with all the groups; t_{clas} – time of classifying the reads to the best group. Bold values indicate the best score.

Table 3. Classification results for the MetaPhyler simulated metagenomic data set (66 841 reads, 300 bp) obtained using CoMeta *allDb*

k	TP	FP	NC	Sensitivity [%]	Precision [%]	Classified [%]	t_{com} [hh:mm:ss]	t_{clas} [hh:mm:ss]	t_{all} [hh:mm:ss]	Memory [MB]
15	50999	15873	0	76.26	76.26	100	00:38:19	00:00:11	00:38:30	5508
	50999	15873	0	76.26	76.26	100	00:38:19	00:00:20	00:38:39	5508
18	59775	7097	0	89.39	89.39	100	00:59:03	00:00:03	00:59:06	48363
	59775	7097	0	89.39	89.39	100	00:59:03	00:00:12	00:59:15	48363
21	66521	353	0	99.47	99.47	100	00:50:23	00:00:02	00:50:25	67342
	66521	353	0	99.47	99.47	100	00:50:23	00:00:11	00:50:34	67342
24	66625	247	0	99.63	99.63	100	00:13:16	00:00:02	00:13:18	70743
	66625	247	0	99.63	99.63	100	00:13:16	00:00:10	00:13:26	70743
**27**	66634	240	0	99.64	99.64	100	00:34:42	00:00:02	00:34:44	73018
	**66634**	**240**	**0**	**99.64**	**99.64**	**100**	00:34:42	00:00:11	00:34:53	73018
30	66628	246	0	99.63	99.63	100	01:06:37	00:00:02	01:06:39	75794
	66628	246	0	99.63	99.63	100	01:06:37	00:00:09	01:06:46	75794

The first row is for the classification without taking into account the *mismatch* file, the second row takes it in into account. t_{com} – time of comparing all the reads with all the groups; t_{clas} – time of classifying the reads to the best group. Bold values indicate the best score.

Table 4. Classification results for the CARMA 454 simulated metagenomic data set (25 000 reads, 265 bp) obtained using CoMeta *allDb*

k	TP	FP	NC	Sensitivity [%]	Precision [%]	Classified [%]	t_{com} [hh:mm:ss]	t_{clas} [hh:mm:ss]	t_{all} [hh:mm:ss]	Memory [MB]
15	15454	10148	0	60.36	60.36	100.00	00:10:09	00:00:08	00:10:17	5182
	15454	10148	0	60.36	60.36	100.00	00:10:09	00:00:16	00:10:25	5182
18	22122	2965	0	88.18	88.18	100.00	00:28:28	00:00:02	00:28:30	49203
	22122	2965	0	88.18	88.18	100.00	00:28:28	00:00:19	00:28:47	49203
**21**	24951	203	2	99.19	99.19	99.99	00:24:29	00:00:01	00:24:30	66243
	**24952**	**203**	**1**	**99.19**	**99.19**	**100.00**	00:24:29	00:00:12	00:24:41	66243
24	24950	218	20	99.06	99.13	99.92	00:27:47	00:00:02	00:27:49	71313
	24955	218	15	99.07	99.13	99.94	00:27:47	00:00:12	00:27:59	71313
27	24869	215	106	98.73	99.14	99.58	00:17:03	00:00:01	00:17:04	69175
	24886	215	89	98.79	99.14	99.64	00:17:03	00:00:11	00:17:14	69175
30	24667	213	307	97.94	99.14	98.77	00:17:49	00:00:01	00:17:50	75913
	24736	215	237	98.21	99.14	99.05	00:17:49	00:00:12	00:18:01	75913

The first row is for the classification without taking into account the *mismatch* file, the second row takes it in into account. t_{com} – time of comparing all the reads with all the groups; t_{clas} – time of classifying the reads to the best group. Bold values indicate the best score.

Table 5. Classification results for the PhyloPythia 961 simulated metagenomic data set (114 457 reads, 961 bp) obtained using CoMeta *allDb*

k	TP	FP	NC	Sensitivity [%]	Precision [%]	Classified [%]	t_{com} [hh:mm:ss]	t_{clas} [hh:mm:ss]	t_{all} [hh:mm:ss]	Memory [MB]
15	64614	48654	1189	56.45	57.05	98.96	00:13:10	00:04:57	00:18:07	4659
	64661	49077	719	56.49	56.85	99.37	00:13:10	00:05:01	00:18:11	4659
18	88632	15152	10673	77.44	85.40	90.68	01:20:12	00:05:00	01:25:12	19000
	88964	22349	3144	77.73	79.92	97.25	01:20:12	00:04:47	01:24:59	19000
21	106743	521	7193	93.26	99.51	93.72	01:20:42	00:04:11	01:24:53	20269
	107538	1685	5234	93.95	98.46	95.43	01:20:42	00:04:49	01:25:31	20269
24	106739	405	7313	93.26	99.62	93.61	00:51:33	00:03:38	00:55:11	23026
	107671	734	6052	94.07	99.32	94.71	00:51:33	00:03:45	00:55:18	23026
27	106635	351	7471	93.17	99.67	93.47	00:47:02	00:03:31	00:50:33	20790
	**107665**	**552**	**6240**	**94.07**	**99.49**	**94.55**	00:47:02	00:03:34	00:50:36	20790
30	106533	302	7622	93.08	99.72	93.34	00:33:18	00:04:28	00:37:46	20330
	107613	460	6384	94.02	99.57	94.42	00:33:18	00:04:34	00:37:52	20330

The first row is for the classification without taking into account the *mismatch* file, the second row takes it in into account. t_{com} – time of comparing all the reads with all the groups; t_{clas} – time of classifying the reads to the best group. Bold values indicate the best score.

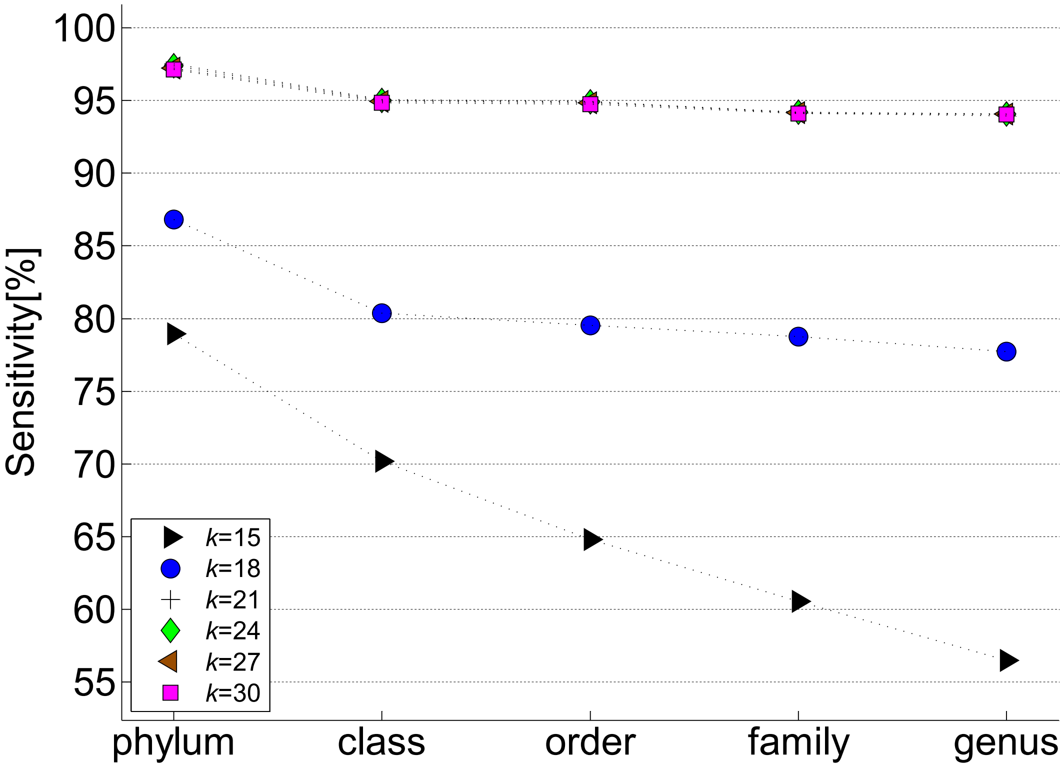

Figure 1. The classification sensitivity obtained for the *PhyloPythia 961 bp* data set using CoMeta *allDb*. The graph shows how the sensitivity varies with the classification to subsequent branches in the taxonomic tree for different lengths of k-mer (k).

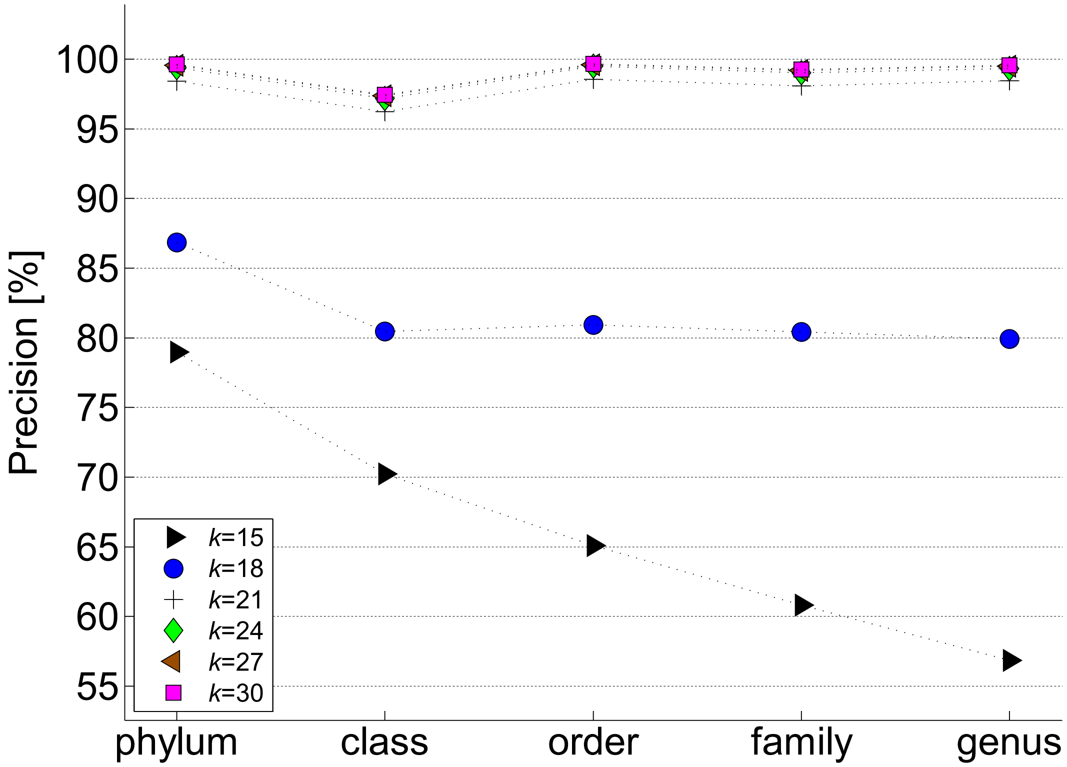

Figure 2. The classification precision obtained for the *PhyloPythia 961 bp* data set using CoMeta *allDb*. The graph shows how the precision varies with the classification to subsequent branches in the taxonomic tree for different lengths of k-mer (k).

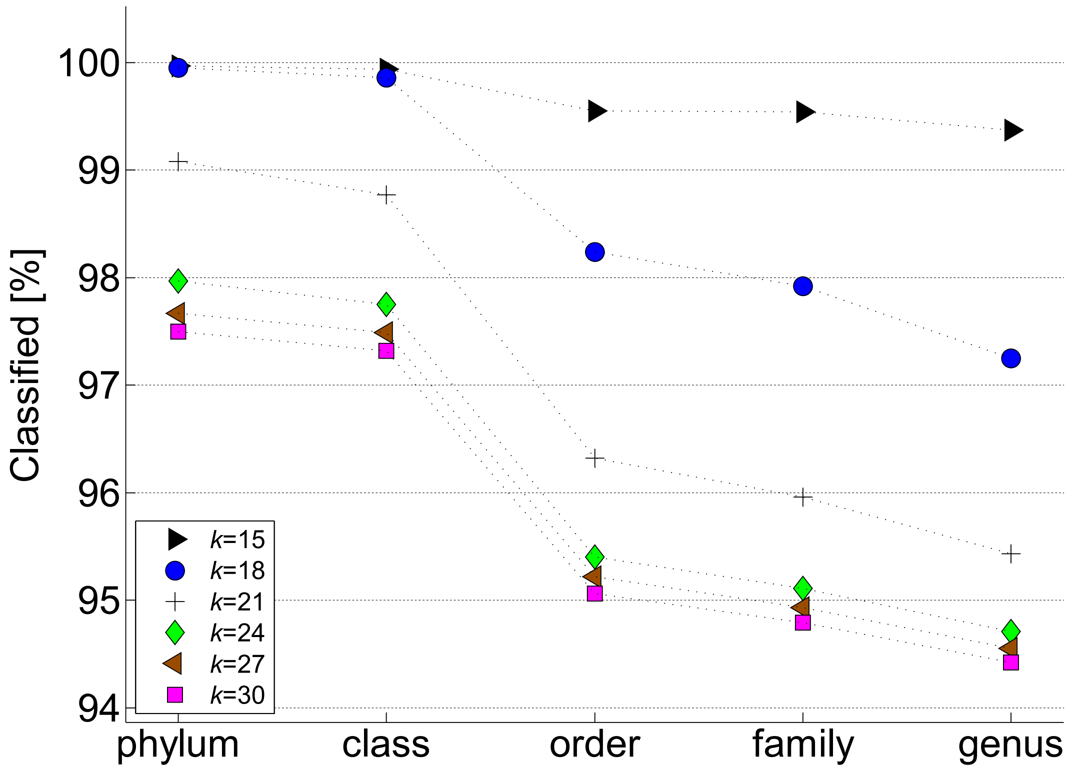

Figure 3. The number of classified reads obtained for the *PhyloPythia 961 bp* data set using CoMeta *allDb*. The graph shows how the percentage of classified reads varies with the classification to subsequent branches in the taxonomic tree for different lengths of k-mer (k).

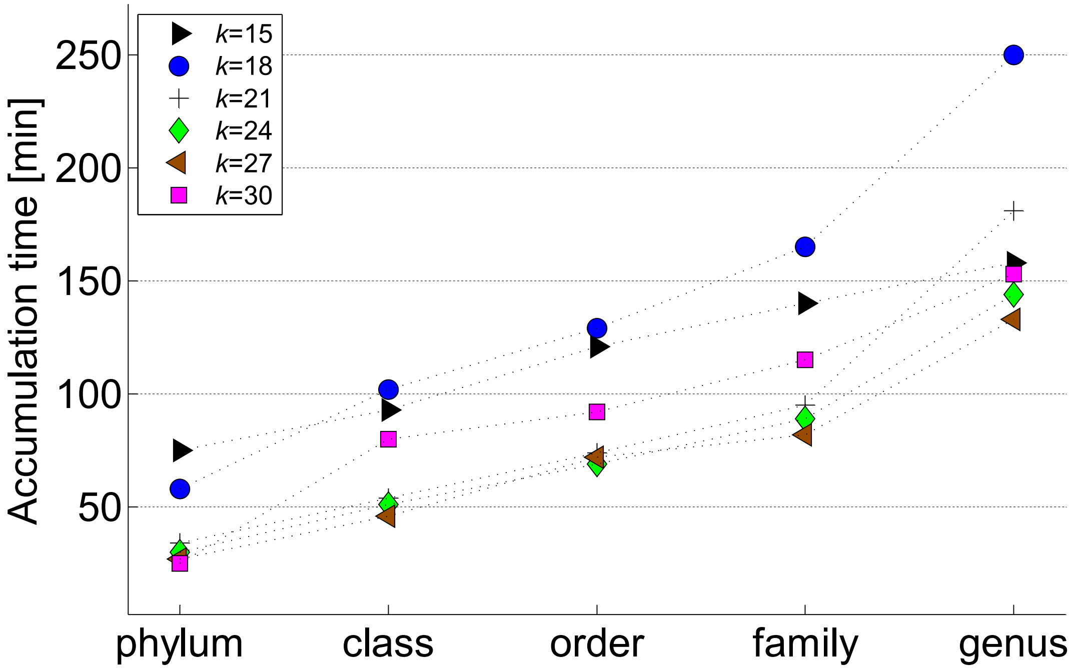

Figure 4. Accumulated processing times obtained for the *PhyloPythia 961 bp* data set using CoMeta *allDb*.

1.2 LMAT

We examined the LMAT algorithm to select the best parameters to compare this program with CoMeta. We checked the classification results for the two databases built by the LMAT authors (*Db*: *kML* and *kFull*), as well as using two values of the "minimum score" (*ms*: 0 and 1)—LMAT parameter, which is responsible for distantly related between read and reference group (when read is assigned to the taxonomic label). The classification results (see Table 6) show that for $ms = 0$, the number of all classified sequences is highest, unfortunately, besides the number of correctly classified, also the number of incorrectly classified increases. The size difference in the number of classified reads is not constant, it depends on which k-mer database and set of reads was used. We expected that for larger database, more reads should be correctly classified, but this was not observed for the *PhyloPythia 961 bp* set. For this set of reads, the classification results obtained using the *kML* database were better. Probably because of the fact that this set was classified to a lower level (genus), where k-mer frequently appearing are more significant, and *kML* is the marker database that contains only k-mers repeated not less than 1000 times. The *FACS* set contains human chromosome reads and *kML* database does not contain k-mers derived from chordates, while *kFull* already contains them. Therefore, we also showed the classification results when reads of chordates are not taken into account using *kML* database (annotation: ^{a}).

Table 6. Classification results obtained using LMAT

Db	ms	TP	FP	NC	Sensitivity [%]	Precision [%]	Classified [%]	t_{clas} [hh:mm:ss]	t_{an} [hh:mm:ss]	t_{all} [hh:mm:ss]
CARMA 454 simulated metagenomic data set (25 000 sequences. 265 bp)										
kML	0	20108	10	4882	80.43	99.95	80.47	00:40:21	00:02:34	00:42:55
	1	13209	9	11782	52.84	99.93	52.87	00:40:21	00:05:26	00:45:47
**kFull**	**0**	**21501**	**9**	**3490**	**86.00**	**99.96**	**86.04**	00:34:49	00:02:44	00:37:33
	1	17	0	24983	0.07	100.00	0.07	00:34:49	00:02:11	00:37:00
MetaPhyler simulated metagenomic data set (66 841 sequences. 300 bp)										
kML	0	63987	1462	1392	95.73	97.77	96.50	00:57:21	00:00:48	00:58:09
	1	51331	1367	14143	76.80	97.41	78.84	00:57:21	00:17:25	01:14:46
**kFull**	**0**	**65859**	**1473**	**458**	**98.53**	**97.81**	**99.31**	03:14:41	00:18:22	03:33:03
	1	61610	1415	3816	92.17	97.75	94.29	03:14:41	00:49:22	04:04:03
Reduced FACS simHC metagenomic data set (93 653 sequences. 269 bp)										
kML	0	24653	30	68970(2366[a])	26.32(91.14[a])	99.88	26.36(91.25[a])	00:59:20	00:00:48	01:00:08
	1	17866	27	75760(9156[a])	19.08(66.05[a])	99.85	19.11(66.15[a])	00:59:20	00:04:08	01:03:28
**kFull**	**0**	**92478**	**16**	**1159**	**98.75**	**99.98**	**98.76**	01:28:19	00:04:46	01:33:05
	1	38	0	93615	0.04	100.00	0.04	01:28:19	00:02:58	01:31:17
PhyloPythia simulated metagenomic data set (114 457 sequences. 961 bp)										
**kML**	**0**	**112281**	**208**	**1968**	**98.10**	**99.82**	**98.28**	05:36:33	00:11:29	05:48:02
	1	110844	191	3422	96.84	99.83	97.01	05:36:33	00:14:10	05:50:43
kFull	0	94451	193	19813	82.52	99.80	82.69	09:38:57	02:23:56	12:02:53
	1	1735	3	112719	1.52	99.83	1.52	09:38:57	00:50:23	10:29:20

[a] – classification results obtained if the reads derived from a human chromosome are not taken into account.
Db – database used for classification.
ms – value of minimum score in LMAT algorithm when assigning a taxonomic label to the read.
Bold values indicate the best score.

2 Database building

The k-mer databases consist of all reference sequences downloaded from the NCBI website. The overall sizes of the databases for classification at the phylum rank are presented in the main paper. Sizes for each non-compact database, that are loaded during the "Comparison" step, are provided in Tables 7 to 9. The largest set of k-mers is for the "Chordata" phylum (up to 73 GB for $k = 30$). For bacteria, the Proteobacteria k-mer database is the largest one, and we need almost 20 GB of RAM memory for comparison with this set.

Figure 5 shows the dependence of the database size on the number of distinct k-mers (which appeared at least once in the *gi* group).

Table 7. ***k*-mer non-compact database size (part 1/3)**

Phylum	$k = 15$	$k = 18$	$k = 21$	$k = 24$	$k = 27$	$k = 30$
			Archaea			
Crenarchaeota	492M	538M	545M	550M	554M	558M
Euryarchaeota	1.2G	1.5G	1.6G	1.6G	1.6G	1.6G
Korarchaeota	13M	13M	13M	13M	13M	13M
Nanoarchaeota	4.4M	4.4M	4.4M	4.4M	4.5M	4.5M
Thaumarchaeota	82M	85M	87M	88M	89M	90M
undef	87M	97M	104M	112M	119M	125M
			Bacteria			
Acidobacteria	328M	358M	361M	363M	365M	367M
Actinobacteria	1.9G	4.5G	5.2G	5.4G	5.4G	5.5G
Aquificae	112M	117M	118M	118M	119M	119M
Armatimonadetes	1.1M	1.2M	1.2M	1.3M	1.3M	1.4M
Bacteroidetes	1.7G	2.3G	2.3G	2.3G	2.3G	2.3G
Caldiserica	13M	13M	13M	13M	13M	13M
candidate division EM 3	528K	528K	528K	528K	528K	528K
candidate division WPS-1	536K	536K	536K	536K	536K	536K
candidate division WPS-2	540K	540K	540K	540K	540K	540K
candidate division ZB3	540K	540K	540K	540K	544K	544K
Chlamydiae	158M	166M	168M	169M	171M	172M
Chlorobi	206M	215M	217M	218M	218M	219M
Chloroflexi	353M	384M	389M	393M	395M	398M
Chrysiogenetes	23M	23M	23M	23M	23M	23M
Cyanobacteria	954M	1.2G	1.2G	1.2G	1.2G	1.2G
Deferribacteres	76M	79M	79M	79M	79M	79M
Deinococcus-Thermus	278M	341M	349M	352M	354M	356M
Dictyoglomi	29M	29M	30M	30M	30M	30M
Elusimicrobia	22M	23M	23M	23M	23M	23M
Fibrobacteres	31M	31M	31M	31M	31M	31M
Firmicutes	3.3G	6.0G	6.3G	6.4G	6.5G	6.6G
Fusobacteria	96M	107M	108M	108M	108M	109M
Gemmatimonadetes	38M	39M	39M	39M	40M	40M
Ignavibacteria	52M	53M	54M	54M	54M	54M
Lentisphaerae	972K	1.1M	1.1M	1.1M	1.2M	1.2M
Nitrospirae	73M	74M	74M	75M	75M	75M
Planctomycetes	286M	305M	308M	310M	312M	313M
Poribacteria	892K	904K	916K	928K	940K	948K
Proteobacteria	5.3G	16G	18G	19G	19G	19G
Spirochaetes	564M	653M	667M	675M	682M	687M
Synergistetes	79M	80M	81M	81M	81M	82M
Tenericutes	218M	276M	287M	291M	295M	299M
Thermodesulfobacteria	30M	31M	31M	31M	31M	31M
Thermotogae	202M	217M	218M	220M	220M	221M
undef	611M	765M	878M	983M	1.1G	1.2G
Verrucomicrobia	116M	120M	121M	122M	122M	123M

Table 8. k-mer non-compact database size (part 2/3)

Phylum	$k = 15$	$k = 18$	$k = 21$	$k = 24$	$k = 27$	$k = 30$
			Eukaryota			
Acanthocephala	2.1M	2.2M	2.3M	2.4M	2.4M	2.5M
Annelida	55M	60M	63M	67M	69M	71M
Apicomplexa	1.2G	1.5G	1.6G	1.6G	1.6G	1.6G
Arthropoda	3.4G	5.8G	6.2G	6.4G	6.5G	6.7G
Ascomycota	4.6G	8.9G	9.2G	9.3G	9.4G	9.4G
Aurearenophyceae	572K	572K	572K	572K	572K	572K
Bacillariophyta	283M	296M	298M	299M	299M	300M
Basidiomycota	1.3G	1.5G	1.6G	1.6G	1.6G	1.6G
Blastocladiomycota	2.4M	2.5M	2.5M	2.6M	2.6M	2.6M
Bolidophyceae	672K	676K	684K	688K	692K	696K
Brachiopoda	2.9M	2.9M	3.0M	3.0M	3.1M	3.1M
Bryozoa	4.9M	5.2M	5.5M	5.7M	5.8M	5.9M
Chaetognatha	2.2M	2.2M	2.3M	2.4M	2.4M	2.5M
Chlorophyta	827M	1.1G	1.1G	1.1G	1.2G	1.2G
Chordata	6.5G	50G	65G	69G	71G	73G
Chromerida	2.6M	2.6M	2.6M	2.6M	2.6M	2.6M
Chytridiomycota	6.4M	6.8M	7.1M	7.3M	7.5M	7.7M
Cnidaria	362M	398M	404M	408M	412M	415M
Cryptomycota	716K	724K	732K	736K	740K	744K
Ctenophora	3.4M	3.4M	3.4M	3.4M	3.4M	3.4M
Cycliophora	596K	600K	604K	604K	608K	612K
Echinodermata	420M	461M	469M	474M	478M	482M
Entoprocta	1.2M	1.2M	1.2M	1.2M	1.2M	1.2M
Euglenida	14M	15M	16M	16M	16M	17M
Eustigmatophyceae	1.2M	1.2M	1.2M	1.3M	1.3M	1.3M
Gastrotricha	1.8M	1.9M	2.0M	2.1M	2.1M	2.2M
Glomeromycota	20M	22M	23M	24M	26M	27M
Gnathostomulida	812K	828K	844K	856K	864K	876K
Haplosporidia	836K	860K	880K	896K	912K	928K
Hemichordata	188M	197M	198M	199M	200M	200M
Kinorhyncha	628K	632K	636K	640K	644K	648K
Loricifera	540K	540K	540K	540K	540K	540K
Microsporidia	112M	118M	119M	120M	120M	120M
Mollusca	174M	197M	210M	221M	232M	240M
Myzostomida	976K	1004K	1.0M	1.1M	1.1M	1.1M
Nematoda	1.2G	1.7G	1.7G	1.8G	1.8G	1.8G
Nematomorpha	748K	756K	764K	772K	780K	784K
Nemertea	4.1M	4.5M	4.7M	5.0M	5.2M	5.3M
Neocallimastigomycota	3.7M	4.4M	5.1M	5.8M	6.6M	7.4M
Onychophora	2.5M	2.6M	2.7M	2.8M	2.9M	2.9M
Orthonectida	536K	536K	540K	540K	540K	540K

Table 9. ***k*-mer non-compact database size (part 3/3)**

Phylum	$k = 15$	$k = 18$	$k = 21$	$k = 24$	$k = 27$	$k = 30$
			Eukaryota contd.			
Phaeophyceae	14M	15M	17M	18M	19M	20M
Picobiliphytes	568K	572K	576K	576K	580K	584K
Pinguiophyceae	812K	824K	832K	840K	848K	852K
Placozoa	115M	119M	119M	119M	119M	119M
Platyhelminthes	1.2G	1.8G	1.9G	1.9G	1.9G	2.0G
Porifera	131M	137M	139M	141M	142M	143M
Priapulida	1.2M	1.2M	1.2M	1.2M	1.2M	1.2M
Rhombozoa	1.2M	1.2M	1.2M	1.2M	1.3M	1.3M
Rotifera	19M	20M	21M	21M	22M	22M
Streptophyta	5.1G	15G	17G	18G	19G	19G
Tardigrada	2.5M	2.7M	2.8M	2.9M	2.9M	3.0M
undef	2.7G	4.3G	4.5G	4.6G	4.7G	4.8G
Xanthophyceae	3.5M	3.6M	3.7M	3.9M	4.0M	4.0M
Xenoturbellida	896K	904K	908K	912K	916K	920K
			Viroids			
undef	1.1M	1.1M	1.2M	1.3M	1.4M	1.4M
			Viruses			
undef	1.1G	1.4G	1.5G	1.6G	1.7G	1.8G

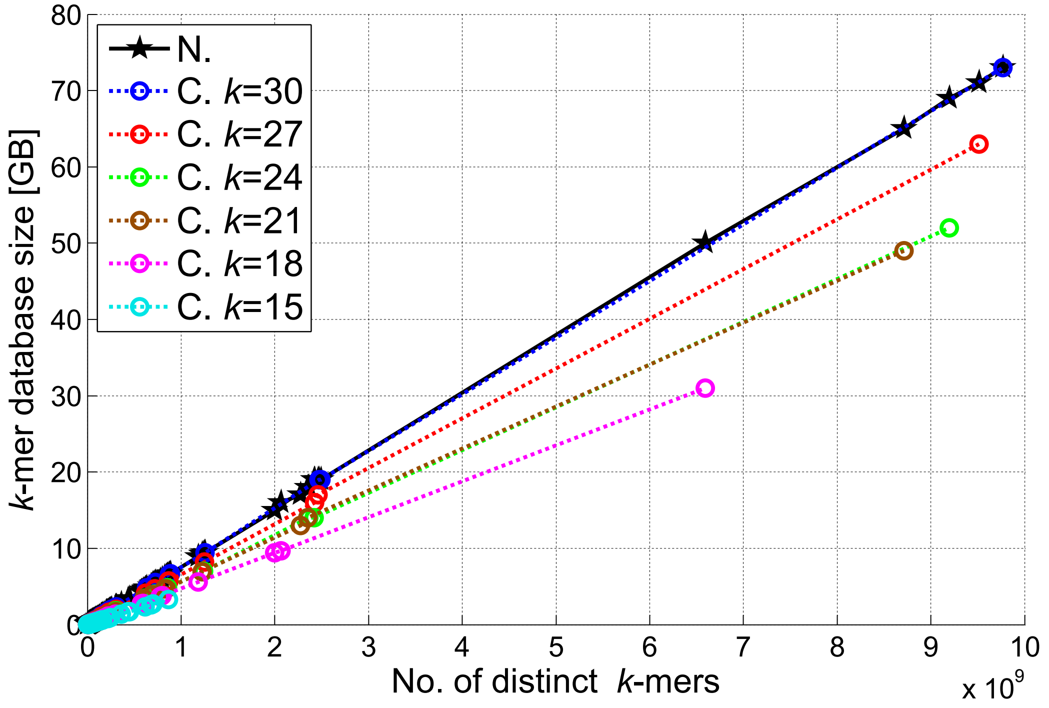

Figure 5. The dependence between the k-mer database size and the number of distinct k-mers. The symbol "N" ("star" mark) represents the non-compact database, whose size does not depend on the length of the k-mers, while the symbol "C" ("circle" mark) represents the compact databases using different lengths of k-mer.
